# Hepatitis C virus genotype 3a with phylogenetically distinct origin is circulating in Pakistan

**DOI:** 10.1186/1479-0556-9-2

**Published:** 2011-01-06

**Authors:** Irshad-ur Rehman, Muhammad Idrees, Muhammad Ali, Liaqat Ali, Sadia Butt, Abrar Hussain, Haji Akbar, Samia Afzal

**Affiliations:** 1 Division of Molecular Virology, National Centre of Excellence in Molecular Biology, University of the Punjab, 87-West Canal Bank Road Thokar Niaz Baig Lahore-53700, Pakistan

## Abstract

**Background:**

Hepatitis C virus (HCV) is one of the leading causes of viral hepatitis worldwide and its genotype 3a is predominant in vast areas of Pakistan.

**Findings:**

The present study reports the first full sequence of HCV 3a isolate PK-1 from Pakistan. This nucleotide sequence was compared with six other HCV genotype 3a full length sequences from different regions of the world by using statistical methods of phylogenetic analysis.

**Conclusion:**

The nucleotide difference of these seven sequences shows that HCV genotype 3a of phylogenetically distinct origin is circulating in Pakistan.

## Findings

Hepatitis C virus (HCV) is leading cause of chronic liver disease [1] with estimated 170-200 million infected persons worldwide [2] including approximately 17 million in Pakistan [3]. It is positive single stranded RNA virus first isolated in 1819 and is a member of *Flaviviridae *[2,4]. The HCV genome is about 9.6 kb in length consisting of single open reading frame encoding a polyprotein of 3,000 amino acids and non-translated regions located at the 5'and 3' terminus [5].

The relative prevalence of HCV genotypes varies with the geographic area but genotypes 1, 2 and 3 have worldwide distribution. 1a and 1b are the most widespread genotypes in the Europe, [6] USA, [7] and Japan [8]. HCV subtype 3a is the most common genotype circulating in India [9], Nepal [10] and Pakistan [11]. HCV genotype 4 prevailing in the Middle East and North Africa [12], and genotypes 5 and 6 appears to be most common to South Africa and Hong Kong, respectively [13]. Genetic analysis of HCV genotype 3a is very important as it is very sensitive to interferon therapy compared to the other genotypes.

Butt and colleagues [2] showed that HCV 3a has been the predominant genotype (causing disease in 62% - 70% patients) in Pakistan based on the last ten years data (2000-2009), which shows that this genotype has been successfully spreading in Pakistan. However, it has not been well characterized genomically. For this purpose, serum sample from patient infected with HCV was obtained and consensus sequence of HCV genotype 3a isolate PK-1 was determined from cDNA using various modified methods [14,15]. In the present study, we report the first full sequence of HCV isolate PK-1 (9474 nucleotides) from Pakistan. This genomic sequence is phylogenetically distinct from the HCV genotype 3a isolates sequenced in the rest of the countries like USA, New Zealand, Italy, Australia and Germany (Table 1).

**Table 1:** List of the hepatitis C virus genotype 3a sequences used in the analysis.

**S. No**.	**GenBank accession No**.	Country	Strain*
**1**	D17763	New Zealand	2005-NZL1
**2**	X76918	Germany	2005
**3**	D28917	USA	2005-HCV-K3a/650
**4**	AF046866	Australia	2007-CB
**5**	Gu294484/Pk	Pakistan	2010-PK-1
**6**	GU814263	Italy	2010-S52
**7**	GU814264	Italy	2010-S52 (Synthetic construct)

*Year of publication of the genome in GenBank and strain if assigned.

GenBank accession numbers, place of isolation, isolate name and publishing year are also mentioned.

Phylogenetic analysis of the isolate PK-1 and all the full length HCV genotype 3a genomes (n = 7) in the GenBank database (Table 1) by using MEGA4 software package. Two different methods (UPGMA method and Neighbor Joining (NJ) method) of the phylogenetic analysis were used as described previously [16]. Evolutionary distances were also estimated by using methods previously reported by Tamura *et al *[16].

**Figure 1:**
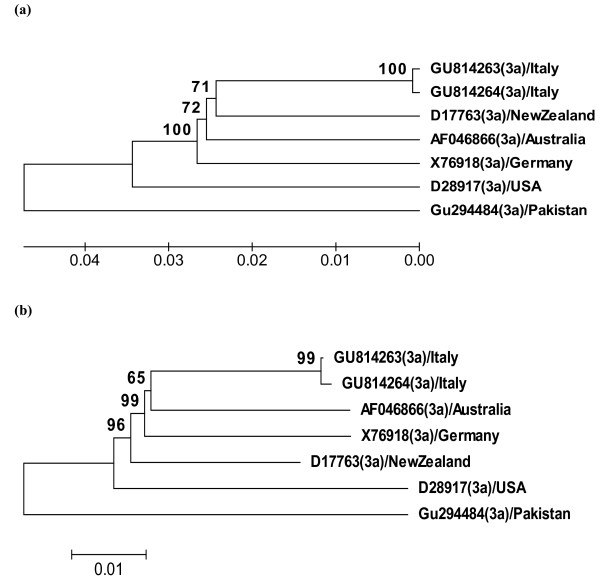
**The bootstrap original phylogenetic tree of hepatitis C virus (HCV) genotype 3a isolates from different regions of the world based on complete genome sequences inferred from 500 replicates representing the evolutionary relationship**. Branches corresponding to partitions reproduced in less than 50% bootstrap replicates are clustered together while; branch lengths are measured by the scale in the same units as those of the evolutionary distances. GenBank accession numbers, genotype and place of isolation are mentioned for all the isolates. **(a) **UPGMA analysis **(b) **Neighbour-Joining method describing the evolutionary relationship between the HCV full length sequences.

The full length genomic sequences of HCV 3a reported from USA, New Zealand, Italy and Australia were clustered together while Pakistani isolate is shown phylogenetically distinct by 500 replicates bootstrap analysis (Figure 1). Estimates of evolutionary divergence between the sequences (Table 1) shows that the Pakistani isolate have evolutionary distance of 0.085-0.103 to the rest of all the full length sequences however, the average distance between all these sequences is 0.0655 ± 0.0071 (Table 2). The analysis also suggests that PK-1 and American isolate (D28917) are the earliest phylogenetic representatives of the HCV genotype 3a that has been successfully spreading in Pakistan and USA respectively, while the rest of the isolates are their late derivatives spreading in Germany, Italy, New Zealand and Australia. As 3a is the predominant genotype in Pakistan, its sequencing data and evolutionary analysis will help in evaluation and development of new antiviral therapies and possible vaccine development. Moreover, the association of HCV genotype 3a full length nucleotide sequences with the epidemiology, severity of disease and its response to interferon therapy needs to be evaluated.

**Table 2:** Estimates of evolutionary divergence between sequences with standard error estimates.

Sequence 1	Sequence 2	Dist	Std. Err
Gu294484(3a)/Pakistan	D17763(3a)/New Zealand	0.0858383	0.0100783
Gu294484(3a)/Pakistan	GU814263(3a)/Italy	0.0929113	0.0108032
D17763(3a)/New Zealand	GU814263(3a)/Italy	0.0480462	0.0056442
Gu294484(3a)/Pakistan	GU814264(3a)/Italy	0.0945252	0.0110770
D17763(3a)/New Zealand	GU814264(3a)/Italy	0.0492012	0.0058641
GU814263(3a)/Italy	GU814264(3a)/Italy	0.0016043	0.0004740
Gu294484(3a)/Pakistan	AF046866(3a)/Australia	0.0965145	0.0109635
D17763(3a)/New Zealand	AF046866(3a)/Australia	0.0520425	0.0061060
GU814263(3a)/Italy	AF046866(3a)/Australia	0.0499546	0.0059291
GU814264(3a)/Italy	AF046866(3a)/Australia	0.0508833	0.0061427
Gu294484(3a)/Pakistan	X76918(3a)/Germany	0.0945054	0.0110161
D17763(3a)/New Zealand	X76918(3a)/Germany	0.0533185	0.0065799
GU814263(3a)/Italy	X76918(3a)/Germany	0.0518464	0.0066160
GU814264(3a)/Italy	X76918(3a)/Germany	0.0526615	0.0067952
AF046866(3a)/Australia	X76918(3a)/Germany	0.0550679	0.0067483
Gu294484(3a)/Pakistan	D28917(3a)/USA	0.1032085	0.0119522
D17763(3a)/New Zealand	D28917(3a)/USA	0.0673865	0.0078749
GU814263(3a)/Italy	D28917(3a)/USA	0.0666617	0.0075880
GU814264(3a)/Italy	D28917(3a)/USA	0.0676199	0.0077599
AF046866(3a)/Australia	D28917(3a)/USA	0.0704518	0.0080246
X76918(3a)/Germany	D28917(3a)/USA	0.0712210	0.0083935
			

* All the positions containing missing data and gapes were eliminated.

## Competing interests

The authors declare that they have no competing interests.

## Authors' contributions

MI conceived of the study. IR, SB, SA, HA and AH performed the sequencing studies. MA and IR organized the data and drafted the manuscript. SB, LA and AH helped MA in sequence alignment and evolutionary analysis. All the authors read and approved the final manuscript.
